# Man Thou Art Dust: Rites of Passage in Austere Times

**DOI:** 10.1177/0038038516633037

**Published:** 2016-03-15

**Authors:** Deirdre M O’Loughlin, Isabelle Szmigin, Morven G McEachern, Belem Barbosa, Kalipso Karantinou, María Eugenia Fernández-Moya

**Affiliations:** University of Limerick, Ireland; University of Birmingham, UK; University of Salford, UK; University of Aveiro, Portugal; Athens University of Economics and Business, Greece; CUNEF, Madrid, Spain

**Keywords:** austerity, communitas, liminality, male identity, recession, rites of passage

## Abstract

In response to recent calls for further cross-disciplinary research on austerity and a deeper sociological understanding of the impact and aftermath of the economic crisis on individuals and societies, this article builds on extant austerity literature through an exploration of its effects on European men. Informed by theories of liminality and rites of passage, this qualitative investigation examines the experience of austerity from the perspective of 11 men through the three liminal stages of separation, transition and reaggregation and investigates its impact on their identity, responsibilities and expectations. Our findings reveal the negative experiences of alienation and outsiderhood alongside positive experiences of communitas, solidarity and comradeship. The study provides a nuanced understanding of modern male Europeans and their ‘rites of passage’ through austere times.

## Introduction

The recently published *Sociology* special issue on ‘Sociology and the Global Economic Crisis’ and the British Sociological Association conference on ‘Sociology in an Age of Austerity’ underscore the relevance and importance of austerity within the domain of sociology, rendering timely, an investigation of austerity both in sociological terms (Dinerstein et al., 2014) and from a cross-disciplinary perspective (Brown and Spenser, 2014). While for some countries the financial crisis is receding, its aftermath continues to be felt (Dinerstein et al., 2014). Stress and uncertainty prevail in post-recession economies, with self-efficacy and self-confidence severely tested (McGregor, 2011). A number of studies have explored the effects of austerity on various demographic groups with different financial circumstances (e.g. Quelch and Jocz, 2009; Sharma and Alter, 2012), as well as the polarisation of those managing through the recession and those who are not (Cappellini et al., 2014; Petersen, 2013). While studies on the topic of low-income/disadvantaged consumers (Hamilton, 2012; Hamilton and Catterall, 2006) have focused on the so-called ‘fixed state’ of poverty (Elms and Tinson, 2012), less research has examined the middle classes (Cappellini et al., 2014). Similarly, much social research has focused on women in vulnerable situations (Cappellini et al., 2014; Spitzer and Piper, 2014; The Voice Group, 2010; Treanor, 2015), with the male perspective largely neglected.

This research involved an in-depth qualitative study of 11 men from a range of demographics across five European nations. The concept of rites of passage (Van Gennep, 1960 [1906]) is used to examine how European males navigate personal challenges resulting from the recession. We consider how their identity, social, emotional and financial stability are buffeted through Van Gennep’s (1960 [1906]) three stages of liminality. The study contributes to the sociology literature by investigating the liminal nature of male Europeans within the significant, if temporal context of austerity. It also sheds light on the effects of liminality on male identities and responsibilities including the traditional roles of father and male provider. We suggest that, for some, the presence of communitas (Turner, 1974) has aided the journey towards reaggregation while, for others, social and economic alienation or ‘outsiderhood’ has prolonged liminality.

## The Nature of Liminality and Rites of Passage

Liminality is constructed as a new identity state to be contrasted with the normal or previous state (Baker et al., 2005) and is often associated with times of grief or other major life challenges (Kimball, 1960). These significant events have also been conceptualised as ‘turning points’ (Mandelbaum, 1973: 181), ‘epiphanies’ (Denzin, 1989: 17), ‘fateful moments’ (Giddens, 1991: 113) and ‘critical moments’ which Thompson et al. (2002: 339) describe as ‘events […] having important consequences for [individuals’] lives and identities’. Critical moments result in an individual undergoing a process of change or transition, such as boyhood to manhood (Nayak, 2014). Liminality has long been associated with rites of passage (Turner, 1974; Van Gennep, 1960 [1906]). Such transition rites refer to marked changes in a person’s state or social position and consist of three core phases. The first is ‘separation’ involving some kind of removal from the stable identity or ‘an earlier fixed point in the social structure’ (Turner, 1974: 232), often prompted by an external event (Noble and Walker, 1997). The second is the ‘marginal’ stage which describes when the liminars (or passengers) find themselves in an ambiguous, betwixt and between situation which removes them from their past and their future (Van Gennep, 1960 [1906]). This stage includes a substantial degree of instability, which in certain cases is typified by social ambiguity (Cody et al., 2011). Finally, ‘reaggregation’ is the identity that emerges from the state of liminality and may be seen as better (and definitely different) to the previous identity. It is important to note that Turner’s (1974) and Van Gennep’s (1960 [1906]) analyses are placed within an anthropological discussion of cultural traditions where the ritual aspect of liminality is emphasised. Nevertheless, it is a useful structure that has been applied to many situations outside of cultural traditions and rituals (Collinson, 2004; Kimmel, 2008).

The process of liminality has positive and negative implications. On the one hand, from liminality, ‘communitas’ (Turner, 1974) (a bonding over and above formal social bonds) can spontaneously emerge through a comradeship of those involved and can play a significant role in ensuring a smooth transition (Collinson, 2004). However, there can also be ‘outsiderhood’ where the passenger is ‘situationally or temporarily set apart’ (Turner, 1974: 233) from others. Since social participation happens through material consumption, not having the resources for such consumption can lead to ‘social retreat and ultimately reduced social capital’ (Chase and Walker, 2012: 740). Liminality can involve a loss of symbols from a previous time; status and authority may be removed and replaced by a levelling and in some traditions by some kind of ordeal. But levelling also holds the potential for resurgence (Van Gennep, 1960 [1906]). Drawing on rites of passage, we explore how European male participants navigate through the often turbulent journey of austere times.

## Gender and Austerity

The concept of masculinity and masculine identity has been explored across a range of studies (e.g. Alexander, 2003; Bordo, 1999; Connell, 1995; Stern, 2003; Thurnell-Read, 2011) with the literature often considering the male provider role and the working family man as traditional male identities (Ferguson, 2001; Riley, 2003). Other approaches have focused upon extreme forms of masculinity embodied by the lad culture or laddism (Dempster, 2009; Phipps and Young, 2015). Traditional concepts of masculinity have been challenged (Harrison, 2008), calling for a rejection of a hegemonic masculinity embodied by the notion of ‘a man’s man’ to a more pluralistic identity embodying both male and female characteristics (De Visser, 2009: 367). On exposing gender differences and stereotypes, a recent study by Garcia-Calvente et al. (2012) contrasted ways in which men and women perceive health, vulnerability and coping with illness. Highlighting gender inequalities in health, they found that while men hid behind the ‘tough guy’ masculine stereotype, women experienced feelings of vulnerability and exhaustion, but coped better with illness than men. The idea of coping is echoed in recent work examining the presentation of male identity through the recession which suggests a resurgence of the trope of masculinity in ‘crisis’ (Negra and Tasker, 2014). There is a growing body of research that has highlighted the need to contemporise the notion of hegemonic masculinity (Donaldson, 1993; Hearn, 2004; Thurnell-Read, 2011) to reflect changing gender orders and categorisations and recognise a movement towards ‘gender democracy’ (Connell and Messerschmidt, 2005: 829).

Several studies have highlighted the gendered dynamics of the recession and its impact on individuals (Spitzer and Piper, 2014; Treanor, 2015). Spitzer and Piper (2014) explored the particular impact of the crisis on female migrant workers who are more susceptible to economic downturns while Treanor (2015) examined the financial and psychological consequences of the recession for mothers and their children. Cappellini et al.’s (2014) study of Italian women found that female responses to austerity also involved a reinforcement of traditional gender roles. On the other hand, austerity can challenge traditional stereotypes. Part of gender democracy is an acceptance that males are subject to weakness and fallibility in their personal and social lives. Men experiencing high levels of financial stress were found to suffer anxiety and depression and ‘psychosocial ill health’ (Starrin et al., 2009: 283). Keating et al. (2013) examined male and female consumers’ responses during the Irish recession, identifying emotional and behavioural strategies including resignation and creativity to cope with their significantly changed circumstances but did not focus on gender differences in coping strategies. Some have asserted that the recession has been ‘a remorseless assault on the identity of many men’ (Peck, 2010). The foregoing shows that while there has been an increase in academic interest around the impact of austerity, there is a lack of research that focuses specifically on the male response in households.

## Methodology

The study took an interpretive approach (Goulding, 1999) to explore the nature of austerity experienced by 32 Europeans. Face-to-face in-depth interviews were conducted by a team of European researchers in five countries. Drawing on professional, community and personal networks to identify potential participants in each country, a purposive sample (Patton, 1990) was then employed to recruit 32 people from Ireland, UK, Spain, Portugal and Greece across a range of demographics (e.g. age, gender, life-stage and income, including unemployed people). In identifying the sample, we sought participants who might have been affected in some way by the recession. Of this, 11 interviews were conducted with European men (see Table 1). The discussion guide facilitated and explored the effects of austerity on everyday lives in terms of practices around the home, work, shopping and leisure, with participants discussing their lives both prior to and during austerity.

**Table 1.** Profile of male participants.

	Name (pseudonym)	Nationality	Age	Profession/employment	Relationship	Children
1	Robert	Irish	38	Public servant	Married	yes
2	David	Irish	60	Mature student	Separated	yes
3	Derek	UK	64	Self-employed carpenter	Married	yes
4	Manuel	Portuguese	53	Unemployed construction worker	Married	yes
5	Filipe	Portuguese	60	Doctor	Married	yes
6	Luisme	Spanish	27	Part-time student/unemployed	Partner	no
7	Bernado	Spanish	46	Orthopaedic surgeon	Married	yes
8	Pepe	Spanish	34	Entrepreneur	Partner	no
9	Antonio	Spanish	38	Unemployed commercial director	Single	no
10	Yiannis	Greek	38	University academic	Married	yes
11	Nikolaos	Greek	41	Setting up financial advisory business	Married	yes

The interviews which were conducted by six female researchers ranged in length from 45 minutes to 1.5 hours. Drawing on reflexivity debates concerning issues of gender (e.g. Arendell, 1997), the open and subjective nature of the male–female dynamic in our study helped to ‘break down the power relationship between researcher and researched’ (Cotterill and Letherby, 1993: 72). Acknowledging also reflexivity considerations within cross-cultural research (e.g. Easterby-Smith and Malina, 1999: 83) the research team shared country-specific insights through engaging in a reflexive dialogue throughout data collection and analysis, allowing for ‘joint sense-making’ to be achieved.

Interviews were transcribed and, if applicable, translated to English. In accordance with ethical approval guidelines of the lead institution, issues of confidentiality were respected and all participant identities were anonymised. Clarification of translations was ongoing across the six researchers as themes were explored and developed. Interview analysis involved coding and the development of initial themes by individual members of the research team. Subsequently, cross-checking of interview transcripts was completed by all members to facilitate the identification, development and refinement of themes. This led the researchers to engage with theories of rites of passage as key findings related to vulnerability and liminality were identified and are here presented under the themes separation, transition, reaggregation, communitas and outsiderhood.

## Discussion of Findings

Building on previous studies (Allen and Synder, 2009; Anderloni et al., 2012; Chase and Walker, 2012; Treanor, 2015), our analysis of the findings revealed varying levels of financial insecurity and concern experienced through austerity as a result of job loss, underemployment, reduced pay and higher taxes. Participants reported various illustrations of the effects of austerity on them and their families. Often holding the position of sole family provider, an important element to male identity (De Visser, 2009), several participants spoke of the burden of shouldering the financial responsibility for the family and the anxiety of failing in this role. Recognising the significant if temporal impact of the recession on our participants, we applied Van Gennep’s (1960 [1906]) theory of liminality involving the three sequential rites of passage – separation, transition and reaggregation – to examine their journey through austerity and its effect on their identity, responsibilities and expectations.

### Separation

According to Van Gennep (1960 [1906]: 141), those in the separation or preliminal stage are detached from their old life, and often experience a change in their social condition. Yiannis, a 38-year-old Greek academic and father of young twins suffered both salary reductions and higher taxes leading to financial insecurity, forcing him to work long hours. This, coupled with his new role as a father, heightened his sense of duty and he reported feeling a ‘sense of hopelessness, the sense of not feeling certain about anything’. Yiannis was powerless to effect change and struggled to understand ‘what brought us here?’ Used to being in a better financial situation, he confessed his difficulty in ‘finding long-lasting joy in anything’, as he recounted stories of his life before austerity and the responsibility of fatherhood:

If we were at a better financial situation, I think I would try to steal some time to go with Anna for a walk, a stroll followed by coffee. This is what I miss mostly – consuming very simple things […] These are the things I would like to do, if we were better off financially, or if we had less pressure, less rushing around, more optimism, less depression. But we have cut down on all indulgence in this gloomy situation. (Yiannis, 38, Greek, university academic)

He went on to talk about a fundamental shift in his situation and disconnection between who he was and who he had become, both as a father and a man burdened by financial problems. This was manifested in the loss of even small indulgences, of having a coffee and a stroll. Exercise was an important part of his identity but now he found he was constrained by time pressures and guilt at not being more present to help his wife mind his children:

I believe that working with my body was a dialogue, a way to restore balance. It was entrenched in me since I was a child. It was a central part of my identity. Then this changed […] Especially also since Anna is overburdened with the children, it would feel such a selfish thing to do, engaging in an activity that would keep me away from home even longer. The fact that I am away from home so much has been a constant conflict between us because obviously she feels so pressured and tired, so anything I do for myself would seem very selfish. (Yiannis, 38, Greek, university academic)

Yiannis was separated from his previous life, partly through the rite of passage of becoming a new father alongside his increasing workload. These life changes represent ordeals (Turner, 1974) signifying the loss of his previous identity. While bringing home a wage and being a father reflect some of the positive aspects of hegemonic masculinity (Connell and Messerschmidt, 2005), he struggles to succeed at being both breadwinner and involved father (Connell, 1995). In this separation stage, Yiannis was struggling to come to terms with the destabilising effect of austerity and his new role which had shaken his self-efficacy (McGregor, 2011) and impacted his identity. In his struggle to accept his situation, he experienced negativity and hopelessness. Nevertheless in the experience of this rite of passage, Yiannis had recognised his wife’s needs as well as his own.

Other participants reported similar feelings of separation from who they were and the positions they held before austerity changed their lives. Antonio, a 38-year-old Spanish unemployed commercial director compared his previous affluent lifestyle to the new reality of being unemployed, and surviving on a significantly reduced income. He presents a classic representation of a crisis of masculinity created through loss of work (Banet-Weiser, 2014):

There is a before and after; in positions that I was before I earned a lot of money, I invested in buying a second big house, and I bought everything you can imagine […] Now I have incurred these expenses and I need to keep covering them. I had a very good salary and now I’m earning 900 euros from the unemployment office, so I need to reduce everything. I still have to pay the mortgage, phone, expenses […] now I can’t afford it. Before I went out a lot, dinner in restaurants and now much less […] vacation […] nothing. (Antonio, 38, Spanish, unemployed commercial director)

The source of Antonio’s separation was overwhelmingly linked to his reduced financial circumstances; he clearly compared a before and after state. Being unemployed impacted on his identity and perceived social standing, making him feel inferior to what he referred to as his previous middle class status:

It is not a moment of happiness, it’s time to let go […] I feel terrible, worthless, like shit […] you don’t feel good, you don’t feel fulfilled […] before you put on your suit, you go to the company, take the car and go to the city and you feel important and now you get a rucksack, you get into the bus with a lot of different people […] back to class, back to school […] it’s a huge step back […] I used to be middle class but now I am down a level. (Antonio, 38, Spanish, unemployed commercial director)

This separated state has clearly affected his confidence and perceptions of self-worth in terms of his position in society. Turner (1974: 252) refers to Goffman’s ‘levelling and stripping’ of structural status, eradicating a previous social distinction during this process of liminality. Goffman’s (1968: 130) stripping refers to the process that patients went through when entering a psychiatric ward. The analogy of having one’s rights and liberties removed such that the patient is ‘stripped of almost everything’ reflects the losses that Antonio has experienced. In the psychiatric example the levels could be real with graded living arrangements and similarly, Antonio knows that there are material consequences to his situation, such as relinquishing his car for public transport. For men like Antonio, being in a respected work role is closely bound with identity, and a loss of job implies a significant demotion from power and status, generally associated with the male image (De Visser, 2009), leading to feelings of psychological vulnerability and weakness. Antonio represents Turner’s (1974: 233) outsider who is ‘situationally and temporarily set apart’ from others.

### Transition

According to Turner (1974: 232), the transition, marginal or liminal stage in the rite of passage involves ‘being in a tunnel’ striped of status and authority and removed from social structure. The individual is levelled to a homogenous social state through ‘discipline and ordeal’ (Turner, 1974: 259). Younger participants in this transition stage may struggle to leave their parents’ home and often have to postpone plans to get married and have children. They face longer-term unemployment, emigration or at best lowly paid employment. Luisme expressed his despondency about how he still lived with his parents making no economic contribution to the household:

My parents, they deal with those duties. I do not go shopping for food. I don’t contribute financially to the house […] If I accept a low salary, I could find a job tomorrow, but if I’m looking for a better salary I might [have to] leave Spain, or wait 5–10 years, when the situation will improve. The situation will take a long time to get better. (Luisme, 27, Spanish, part-time student/unemployed)

Luisme represents many young Europeans in austerity-burdened economies unable to make the transition to the next stage of their life and financial independence, unless accepting a lower salary than anticipated. While enduring this liminal state, Luisme chose to study and postpone his life plans:

I am lucky as I got a scholarship but I think the government is not doing enough for people and does not care about those people who need most help with their lives and mortgages, with children […] it has affected me in such decisions as well, I wish I would own a house, have children and get married. I’m thinking of leaving the country if I don’t find a proper job in the next two months. Maybe Canada […] And regarding having children I wish I had children right now, but that costs money because you have to give them an education, and so on. (Luisme, 27, Spanish, part-time student/unemployed)

The only opportunity Luisme saw for his current state to be changed was either to wait for the economy to improve or emigrate, where he thought he might be able to secure a well-paid job. This suggests that, as in many rites of passage, he will be moving on to a very different life, possibly in another country. Luisme’s case reflects a ‘powerlessness that arises from an imbalance in marketplace interactions’ (Baker et al., 2005: 134) and suggests that such transitions may continue for a long time for some people.

While in a very different life-stage, Manuel, a 53-year-old father of two and sole breadwinner, faced limited job opportunities. He did not have a salary he could rely on and his future was uncertain. This in turn obliged him to accept any kind of job, despite poor pay and sub-optimal working conditions, to provide for this family:

I’ll work at anything […] today if you want to work you have to accept whatever they offer you […] I’m working for a month already without any days off […] so I can receive over-time hours and receive a little more money so that I can cover my expenses […] I don’t feel good, I feel forced, as if I don’t have any other alternative […] It is either that or nothing at all. I have to do things that I don’t like and that’s why I feel like I’m being controlled – we have to have money, we have our kids to think of […] I don’t have an alternative because I have a family to care for […] I’m the only one who brings money home. (Manuel, 53, Portuguese, unemployed construction worker)

This importance of being the breadwinner in the narratives of fathers reflects a traditional masculine identity (Brannen and Nilsen, 2006). Manuel spoke at length about the burden of shouldering financial responsibility for the family and the anxiety of failing to provide. Embodying the traditional masculine ideal of the hard working family man (Ferguson, 2001) and male provider (Riley, 2003). Manuel talked about feeling controlled and forced to work long hours with little pay. In this state of transition, he was travelling a path prescribed by his situation and did not know where it would lead. He also anticipated difficulties in funding his children’s education in the future:

I don’t know if I’m going to be able to do what I was planning on doing. My daughter says that she wants to be a doctor and I don’t know if I’m going to be able to pay for her university; I’m scared of not being able to do so […] I’m already thinking about six years from now and if I’m going to be able to do that or not […] Yes, it’s hard. It’s hard because I achieved everything I have with my own work, since I was 12 years old […] I wouldn’t like my kids to say later on that the education they have wasn’t paid for by their parents. (Manuel, 53, Portuguese, unemployed construction worker)

Alongside worries about affording the education he would like for his children, Manuel worried that he would be unable to afford or enjoy the normal activities and experiences available to others such that his children may ‘stop having stories to tell’. In this liminal state, it was his struggle to provide for and realise his plans for his children’s happiness that was at the core of his anxiety. Manuel’s fight to fulfil this all important male provider role, which is bound with traditional masculine identity (Riley, 2003), may result in a loss of his male sense of self and purpose.

### Reaggregation

Reaggregation follows transition where one does not return to ‘normal’ but is gradually able to construct a new identity (Baker et al., 2005) and move to an understanding of self and community (Turner, 1974; Van Gennep, 1960 [1906]). The reaggregation, incorporation or post-liminal rite is the ultimate end point in the rite of passage. While we recognise that all participants may reach this, some seemed to already have moved to a reaggregation state. One participant noted that the nature of his work and life experience had prepared him to adapt to austerity. Derek, a 64-year-old self-employed carpenter with two other part-time businesses described how the ups and downs of his working life have equipped him to be both adaptable and to turn his hand to different jobs:

There’s always been dips and lulls, even back in the good times […] there’s been plenty of troughs and valleys, and throughout my whole working life I’ve just been used to that […] I’m happy to keep on working for as long as I can […] we’re very diverse and […] adaptable to situations whatever they might be in years to come. (Derek, 64, UK, self-employed carpenter)

Not only was Derek versatile in his ability to manage these troughs through several sources of income but he was prepared to continue to work part-time beyond conventional retirement age. He displayed adaptation and creativity (Keating et al., 2013) in this reaggregated stage in which he recognised that conventional retirement may not be possible. It may be that having been through previous rites of passage he was able to adapt, envisioning his future with equanimity.

Others appeared to have learned, accepted and adapted to their changed circumstances and in some cases this had been life changing in a positive way. David was a 60-year-old former businessman who decided to return to full-time study at the height of the recession in Ireland. Single-minded about his decision, he still felt the responsibility of being a parent to his adult daughters. He emphasised that he was optimistic about his future career options and anticipated many working years ahead:

Now, I realise that […] that I am now 60 […] and as you know we’re all going to be living until 140 now […] I will definitely go back to work or I will open my own business. So I made a decision that I was going to be working for at least another 10 years, in other words […] It’s something that I enjoy […] if my health holds out obviously. (David, 60, Irish, mature student)

David travelled through the separation from his previous life as a business man and the liminal stage of being a mature student and was now envisaging his next step. Adapting to student life had made him feel that he was a more rounded person and being connected to young people had brought him something unique and valuable which he hoped to put to good use upon returning to work:

Adapting can be a difficult process […] I felt it was necessary for me to adapt. I was open to, to adapting […] my past work life in relation to working with people I think has helped me […] going back to education as well […] particularly working with younger people – to understand their values, and it’s helped me […] a lot of my basic core values still remain the same but there has been plenty of add-ons with regard to where I am, who I am today […] it has been a process of examining my life in general […] I mean it has given me more time to look at who I am, what I am, where I am and I feel that this has been a process that I’m glad I’ve gone through because I feel that at the end of the day, I’m hoping that I am a more rounded person […] I’ve a lot more to contribute to society in general, including myself both physically and emotionally. (David, 60, Irish, mature student)

Perceiving himself as a survivor of the recession, David appeared optimistic and confident that he could continue to contribute. Adapting to being a mature student during an economic crisis, he had survived a period of liminality (Turner, 1974) and, through a process of self-examination, had emerged with a balanced perspective on life.

While some other participants reached reaggregation through adapting and evolving, Nikolaos’s response to the recession was also proactive, in the sense that he resigned from his job in order to set up his own financial advisory business. While Nikolaos exhibited an entrepreneurial spirit, he was less financially vulnerable than some of the other participants. He was careful to emphasise that having forecast problems in his company he took the risk to set up his business with the view that this would be better for him and his family in the long run:

So a significant part of my decision to resign and try to develop my own business had to do with my forecasting of serious problems emerging in the company I used to work for […] Even though everybody values, myself included, the security of a pay-cheque, I decided that it was best to take my destiny into my own hands and try to build a business that does not rely so much on the Greek economic reality so as to be able to provide greater security in the long run for my family. If this business venture does not work out the way I hope it will, then I have decided that we should leave the country and go abroad. The main driver for this decision is that being in a country as badly hit as Greece is by the economic crisis, will take us down along with everybody else. (Nikolaos, 41, Greek, setting up own financial advisory business)

This review of lifestyle and rationalisation of spending patterns that Nikolaos emphasised was not in response to the crisis but rather what he described as his long-term ‘self-improvement process’. The fact that he considers the possibility of emigration might suggest that he had at least mentally gone through the stages of separation and transition to reach a place of reaggregation (Van Gennep, 1960 [1906]) in terms of what he must do now:

It is a reassessment of lifestyle that is taking place. We consciously try to be more alert to irrational spending in all our activities. The truth is that we have managed to rationalise most spending and make changes, but this is not because of the crisis, it is the result of a long-term effort that we have made. The effort is to rationalise and reassess part of our spending patterns and we consider this part of a self-improvement process […] Because of the crisis, even though it did not have a direct impact on our income before my leaving my job, we have been having a different view on consumption and fairness of prices. (Nikolaos, 41, Greek, setting up own financial advisory business)

Unlike other participants who shared their experiences of liminal vulnerability largely in response to austerity, he acknowledged the effects of austerity on his spending and income but he did not admit to this being the main driver of his rationalisation plan. His response to austerity demonstrates aspects of hegemonic masculinity characterised by dominance, machismo, leadership and competitiveness (De Visser, 2009). As a self-assured alpha male (Connell and Messerschmidt, 2005), he was making deliberate and courageous changes. Having made these difficult choices, adjustments and changes, he may be regarded as reaggregated, actively pursuing his plan for a financially secure future for his family. The establishment of his own business venture represented a creative response to austerity (Keating et al., 2013), albeit a risky one.

### Communitas and outsiderhood

According to Turner (1974: 259), those who are navigating the rites of passage may be stripped of status and authority: much of what has been bound by social structure is ‘liberated, notably the sense of comradeship and communion, in brief, communitas’. While communitas (i.e. a bonding over and above formal social bonds) can spontaneously emerge through a comradeship of those involved, outsiderhood too may be apparent, where the passenger may be ‘situationally or temporarily set apart’ (Turner, 1974: 233). As seen below, our participants provided evidence of both communitas and outsiderhood at various stages of their liminal journeys.

Robert, a 38-year-old Irish public servant, described the social network of friends he has built in his local community. He identified his neighbours as being in a similar position to him and he increasingly drew on them for support and help. This comradeship did not only involve informal social bonds but a sharing of knowledge, skills and materials:

Some things you can’t [fix] […] and you need to call in somebody. Or else checking with your friends to see if anyone has a particular skill in that area […] I’m lucky in that I’ve a good network of Dads in the area […] chances are one of them has been through all this sort of thing before. And that helps an awful lot […] how to fix a leaky shower […] tile a bathroom […] you check with your friends first […] as an example […] I put out the text to three or four friends. I borrowed a friend’s trailer […] that same night; the three of them came over and helped for an hour and a half. You know, we’d have a bottle of wine in the kitchen afterwards […] but there is a good network of people now who are willing to help each other out, whereas I’m not sure that would have been the case before. (Robert, Irish, 38, public servant)

Noting that such spontaneous support may not have occurred before austerity, Robert derived benefits from this communitas which in turn helped him and others in his network cope with their changed situation. This is a particular type of communitas of people with similar experiences not only through austerity but also in their family role, with Robert referring to his ‘network of Dads’.

As the bonds of communitas are often ‘anti-structural […] undifferentiated, equalitarian’ (Turner, 1974: 275), some participants identified with those of a lesser social status, and felt empathy and ‘solidarity’ (Filipe, 60, Portuguese, doctor). While many of our participants felt aggrieved by their own drop in status and security, several shared examples of others whom they perceived as worse off. Bernado, a 46-year-old Spanish surgeon has had to adapt to a significant salary reduction but emphasised that he was still in a privileged position while he felt both sad and close to those who were more severely affected by austerity:

It is so sad, you see the news and I feel so bad. I never imagined this crisis in my life. The rich people are richer, the people with salaries like us are worse and worse off, part of the middle class is disappearing, and the poor people are poorer. So they suffer very big changes […] no house, no food, the children […] I’m so sorry about these situations. And I feel close to them. (Bernado, 46, Spanish, orthopaedic surgeon)

Here, communitas is presented as empathy and concern for the plight of others. In coping with his own rite of passage, Bernado feels a connection to those that are more poorly off than him. Communitas through the shared experience of austerity has brought about a stripping and levelling of structural status (Turner, 1974: 252), bringing people of different backgrounds closer together. Similarly, although Yiannis struggles with the pressures of austerity and fatherhood and feels strain and conflict in his relationship with his wife, he has developed communitas through connecting with others who share similar problems. When a group have a common theme which unites them, as in his case, other issues such as ideological differences may be sublimated:

We influence each other, many decisions, many consumption decisions, you know when you talk with others, you get a sense of their decisions and rationale and you adjust […] adjusting pretty much with what other people do […] we may differ in part because of our ideologies, many of them are in favour of many aspects of the economic policy […] and I am totally, utterly against all of them […] This actually, quite interestingly, does not influence how we behave, how we worry about things and what we do, which is rather similar. (Yiannis, 38, Greek, university academic)

Liminality had, however, resulted in outsiderhood for others. Such outsiderhood took the form of disconnection and alienation from others. This may result in a dislocation or distancing in personal relationships. David also spoke about feeling ‘excluded from his own generation’ as a mature student while Pepe described how his friends ‘don’t call me as before to go out’ (Pepe, 34, Spanish, entrepreneur). Some men, like Pepe, clearly experienced outsiderhood which appeared to be making their rite of passage more difficult to endure. For others, such as Robert and Yiannis, the communitas formed from shared experiences and anxieties of austerity may help transition from liminality to reaggreation and ultimately enable them to experience some form of resurgence (Van Gennep (1960 [1906]).

## Conclusions

Responding to recent calls for further cross-disciplinary research on austerity (Brown and Spenser, 2014) and a deeper sociological understanding of the impact of the economic crisis and its aftermath on individuals and societies (Dinerstein et al., 2014), this article builds on extant austerity literature through an investigation of its impact on European men. Through the application of rites of passage theory, the article has examined the impact on male identities and roles and their responses to austerity. Using Turner’s (1974) and Van Gennep’s (1960 [1906]) three phases of transition as a framework, we have shown how the European men in our study have coped with the rites of passage through austerity. By having participants from five European countries, we have illustrated some commonality in the rites of passage experienced.

Our participants, from diverse demographic and socio-economic backgrounds, displayed individual, social and financial concerns in response to the impact of austerity. In every case, the men have been affected through the experience of austerity in terms of their identity, expectations and aspirations. For some, for example Manuel and Yiannis, this has been a particularly hard road to travel, while others, such as Nikolaos and David, have been better prepared or have taken a pragmatic approach to their situation. Most participants exhibited coping strategies in their presentation of male identity but there were also examples of masculinity in crisis that reflect how the change in the envisioned life course has undermined not only traditional identities of working man, provider and father but also physical and social identities. The article has added to the literature that examines what coping looks like for male identities during austerity.

The application of rites of passage theory shows, particularly for those in the liminal/transition stage, that just as it may take a long time for economies to recover, it will also take time for those affected, to move to reaggregation. While the majority of our participants could not be classed as severely disadvantaged, the levelling caused by a change to their financial circumstances as a result of austerity was perceived as significant and most deeply felt in terms of damage to their identity and a sense of loss of status in their family, community and society. How well they coped with the psychological and sociological effects of austerity along their life trajectory was affected by their ability to connect with similar others and experience a sense of communitas. For some, experiencing outsiderhood and struggling with the separation from their old life, resulted in feelings of exclusion and potential psychosocial ill-health (Starrin et al., 2009). Austerity affected the men in our study not only in regard to their financial stability but in many other aspects of their wellbeing including their self-concept, family and social relations.

Through this exploration of rites of passage, our research reveals that participants often held deep-seated traditional roles of male provider, family man and father which were greatly affected by the recession. The hegemonic masculinity bound with being a ‘manly man’ (Connell and Messerschmidt, 2005; De Visser, 2009) is reflected in some of our participants’ discourse, and within that frame of mind, any change that might negate or reduce this, is difficult for them to deal with. Rather than indicating an accommodation of new roles for men coping with austerity, our study points to a reinforcement of gender stereotyping similar to that identified in Cappellini et al’s. (2014) research on women and austerity, evidenced through the deep engagement by many of our participants with traditional male roles. While the research reveals that the rites of passage may result in positive reaggregation, it is inevitable that the humiliation of what some men have experienced in terms of status and identity will have unsatisfactory outcomes. Therefore, for some, Turner’s (1974: 53) statement ‘Man thou art dust’ rings true.^1^

## Notes

1. Turner (1974: 53) describes the liminal phase of Ndembu rites of passage where communitas is ‘engendered by ritual humiliation, stripping of signs and insignia of preliminal status, ritual levelling and ordeals and tests of various kinds, intended to show that “man thou art dust!”’
